# The relation between global palm distribution and climate

**DOI:** 10.1038/s41598-018-23147-2

**Published:** 2018-03-16

**Authors:** Tammo Reichgelt, Christopher K. West, David R. Greenwood

**Affiliations:** 1 Lamont Doherty Earth Observatory, Columbia University, New York, USA; 2 Geological Sciences, University of Saskatchewan, Saskatoon, Saskatchewan Canada; 3 Biology Department Brandon University, Brandon, Manitoba R7A 6A9 Canada

## Abstract

Fossil palms provide qualitative evidence of (sub-) tropical conditions and frost-free winters in the geological past, including modern cold climate regions (e.g., boreal, or polar climates). The freeze intolerance of palms varies across different organs and life stages, with seedlings in particular less tolerant of sub-zero temperatures than adult plants, limiting successful establishment of populations while permitting adult palms to survive in cultivation outside their natural ranges. Quantitatively, palms indicate minimum cold month mean temperature (CMMT) at 2–8 °C in palaeoclimate reconstructions. These data have accentuated model-proxy mismatches for high latitudes during Paleogene hyperthermals when palms expanded poleward in both hemispheres. We constructed a manually filtered dataset of >20,000 georeferenced Arecaceae records, by eliminating cultivars. Statistically derived mean annual temperature, mean annual temperature range, and CMMT thresholds for the Arecaceae and lower rank subfamilies and tribes reveal large differences in temperature sensitivity depending on lower taxonomic classification. Cold tolerant tribes such as the Trachycarpeae produce thresholds as low as CMMT ≥ 2.2 °C. However, within the palm family, CMMT < 5 °C is anomalous. Moreover, palm expansion into temperate biomes is likely a post-Palaeogene event. We recognize a CMMT ≥ 5.2 °C threshold for the palm family, unless a lower taxonomic rank can be assigned.

## Introduction

Reconstructing climates of the geological past, particular temperature, is of considerable interest for understanding climates under higher than present-day atmospheric CO_2_ levels (*p*CO_2_)^1–4^. The climate modelling community as well as geologists use palaeontological proxy evidence of past annual and seasonal temperatures to refine understanding of future climates due to anthropogenic increased *p*CO_2_^1,3,5^. The occurrence of palm fossils is considered a qualitative proxy for a (sub-) tropical climate owing to living palms’ principally tropical distribution and their lack of anatomical and physiological adaptations at all life stages to survive sustained freezing temperatures^6–9^. More quantitatively, the occurrence of fossil palms has been interpreted as an indicator of frost-free winters with coldest month mean temperatures (CMMT) of ≥ 5 °C^10,11^, although more recent work places this limit at 2.2 °C^12,13^. However, fully constraining the threshold of palm winter-temperature tolerance has been problematic for a variety of reasons, outlined below. Eocene minimum CMMT (CMMTmin) reconstructions based on palms have ranged between 2–8 °C^10,11,13–15^, with chamber experiments suggesting CMMTmin for palms may be 1.5–3 °C higher under *p*CO_2_ >800 ppm than modern palm CMMTmin^16^. The difference between the upper and lower boundaries of this range represents a large discrepancy in the interpretation of terrestrial palaeoclimate^14,17,18^ and poses a challenge for palaeoclimate models^1,3,5^. To reconcile this problem, we executed a comprehensive analysis of the relation between distribution and climate of palms globally.

Relatively high carbon-gain efficiency makes palms competitive tropical rain forest plants^19^, with palms even used to track the biogeographical history of tropical rain forests^20^. Palm-temperature limitations are largely due to the inability of their cells to enter physiological dormancy, but also their typical solitary apical meristem and rich in parenchyma cells^6–9^. However, it is possible to cultivate palms outside of their natural range in more temperate climates, but these cultivars create an unrepresentative view of palm temperature tolerance, as palms in this type of environment; (1) would most likely fail to reproduce under natural circumstances, and (2) are not necessarily subject to the extremes of the environment. Nevertheless, multiple naturally occurring temperate palm species occur. *Rhopalostylis sapida*, *Jubaea chilensis* and *Livistona australis* are the most cold-tolerant species in the Southern Hemisphere, occurring well south of the tropics^21^, whereas in the Northern Hemisphere the most cold-tolerant species are *Trachycarpus fortunei*, *Chamaerops humilis* and *Sabal minor*^7^. These species’ cooler temperature tolerances may be ecological or physiological, and differ on a species-by-species basis^7^. However, our study does not focus on why the temperature limits for these species is different, but rather how temperature affects their distribution, and that of the individual palm tribes. Prior studies of modern palms have shown that climate – principally temperature – is a primary determinant of palm distribution, regionally and globally, with an overprint of biogeographical history for palm tribes, and at the local scale factors such as topography and water availability become important^9,21,22^.

The restricted latitudinal distribution of palms as a potential palaeoclimate proxy was quantified by Greenwood and Wing^11^ who established a minimum mean annual temperature (MAT) for palms of 10 °C, a CMMTmin of 5 °C and an absolute yearly minimum of -10 °C. However, that study relied upon tying palm distribution limits of cold tolerant genera such as *Chamaerops*, *Jubaea*, *Livistona, Rhopalostylis, Sabal* and *Washingtonia* to the nearest weather station with long term temperature data, and so did not capture fine detail in the temperature limits of palms, or differences in cold tolerances of individual palm tribes. The CMMT minimum value of Greenwood and Wing^11^ has been applied in many studies^14,18^. More recently, an adjustment of +1.5–3.0 °C to the CMMTmin >5 °C limit was proposed based on a greater plant frost sensitivity under *p*CO_2_ >800 ppm^16^. Furthermore, Walther *et al*.^12^ studied the expanding range of *Trachycarpus fortunei* in Europe under increasing CMMT’s due to present-day anthropogenic climate change, based on the modern climate range of this species in China, and found that *T. fortunei* required a minimum CMMT ≥ 2.2 °C to successfully reproduce in Swiss forests. Fang *et al*.^23^, however lists the minimum CMMT of *Trachycarpus fortunei* as -3.2 °C, a value rejected by some authors^13^. Moreover, Fang *et al*.^23^ list several other palm species with CMMT’s below 2.2 °C. Fang *et al*.^23^ specify that all cultivated species were removed from consideration, but that cultivated individuals that are native to China were not. The clear deviation of the minimum value listed by Fang *et al*.^23^ of other estimates of palm minimum CMMT’s^13^ argues that inclusion of cultivated individuals confounds the true climatic range of palms.

**Figure 1:**
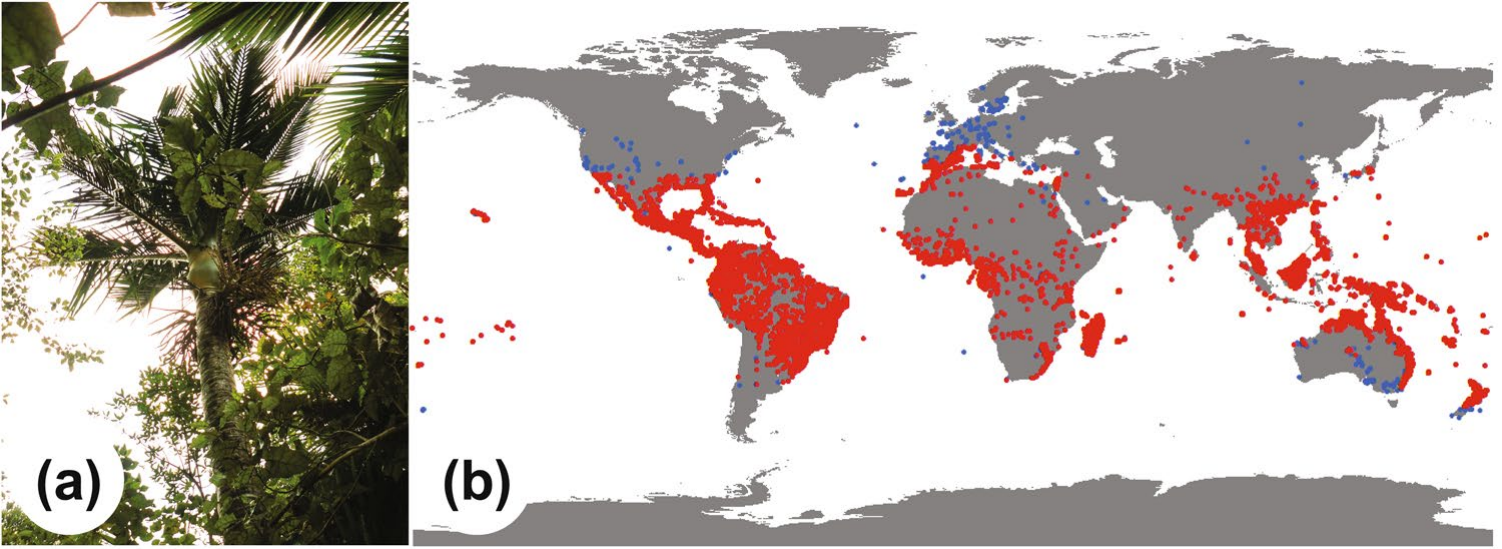
(**a**) Image of *Rhopalostylis sapida*, occurring at the limits of its native range on the West Coast of South Island, New Zealand. (**b**) Recorded geodetic coordinates for palm occurrences worldwide (Supplementary Table 2). Blue circles indicate recorded occurrences that were eliminated by manual filtering and red indicate the occurrences used in this study. Map generated using ArcGIS^58^.

Palms are amongst the most widely cultivated plants in the world. Many palms are popularly cultivated as ornamental plants, as street trees or indoor pot plants. Some palms have been widely planted for their economic importance, such as the African oil palm (*Elaeis guineensis*), the coconut palm (*Cocos nucifera*) and the date palm (*Phoenix dactylifera*). Indeed, cultivation of the date palm may have been as early as 5000 years BP^24^. Because palms have been so widely cultivated, the natural distribution of a single species is often difficult to retrace. This presents a challenge in reconstructing the temperature tolerance threshold of the palm family, as many individuals are artificially maintained in environmental conditions within which they would perish in under natural circumstances (Fig. 1). We approach this problem here by conducting a manual and a statistical filter of the results.

This study focuses on the lower threshold of palm temperature tolerance. We use modern-day palm distribution and rigorously filter and test the climatic conditions at each point to ensure we are capturing a representative range of temperature. Furthermore, a representative climatic tolerance range is determined for each palm tribe. Recent studies of palm classification and phytogeography^25^ provide a phylogenetically robust basis for our analysis, as we focus our study on the various tribes of palms, and the most-cold tolerant members of a smaller set of palm tribes. There are currently 28 palm tribes in five subfamilies recognized^25^. When a palm fossil is assigned to a tribe or even a subfamily, it is likely that the lower taxonomic classification can provide more refined palaeoclimatic constraints. Finally, we will examine the palm threshold as a terrestrial palaeoclimate indicator for several case studies.

## Results

**Figure 2:**
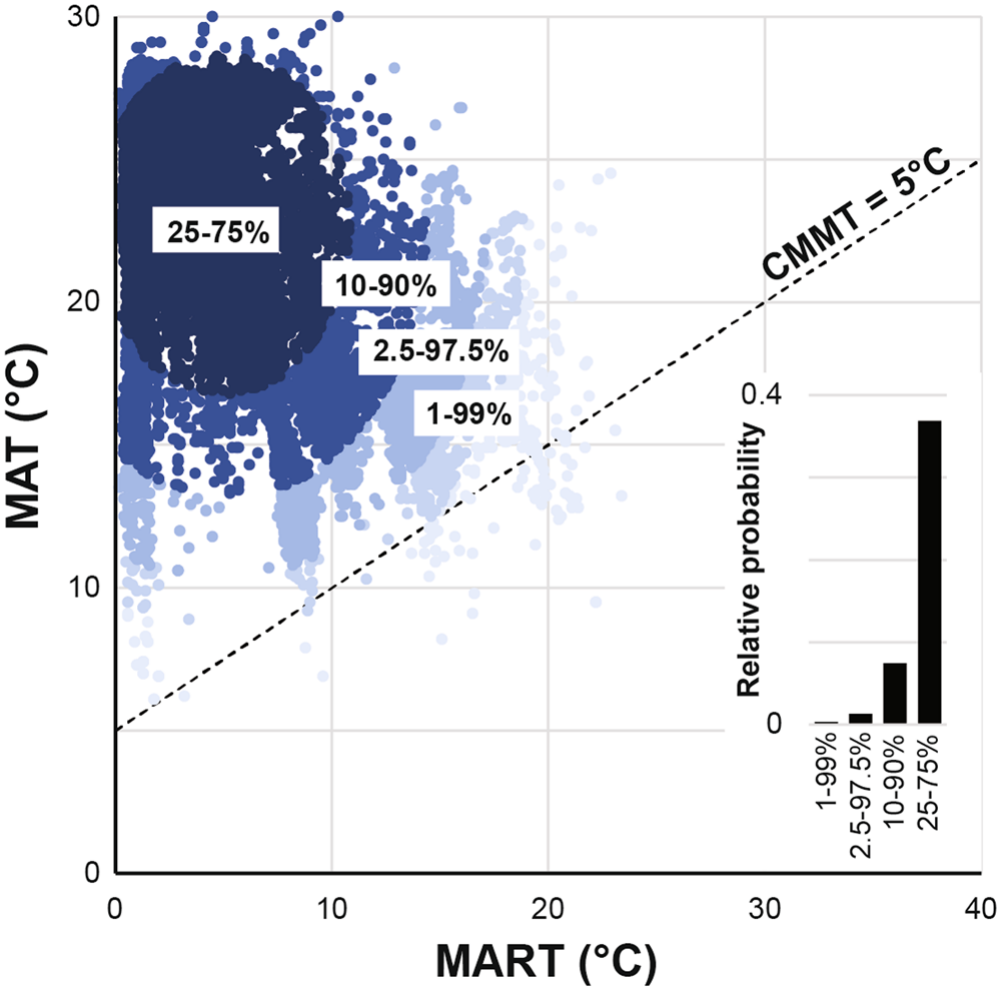
Climatic distribution of Arecaceae, indicating percentile ranges from the core distribution. The bar graph inset indicates the relative probability (Equation 3) that the percentile range constitutes a significant portion of the climatic range within the Arecaceae.

**Figure 3:**
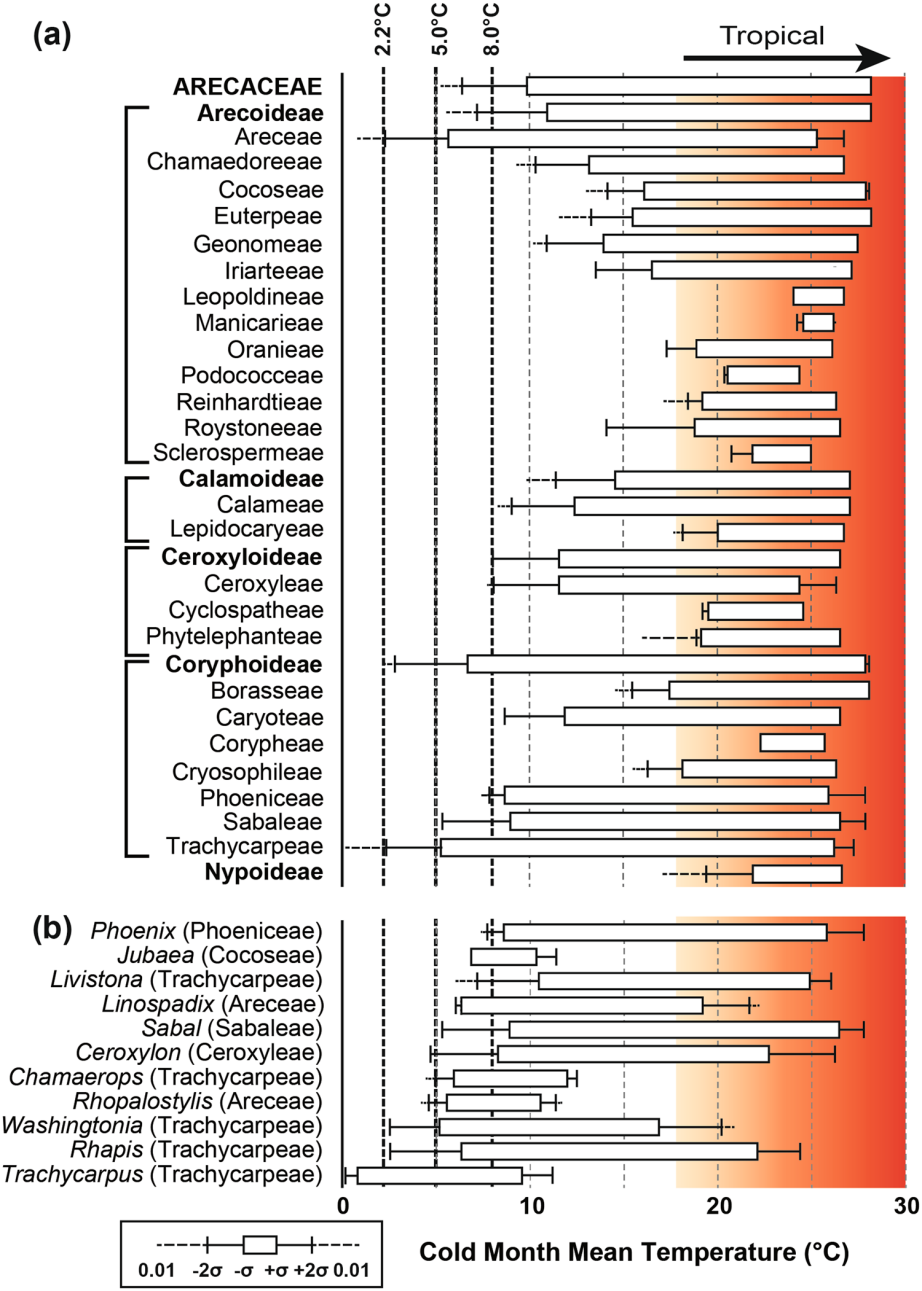
Cold month mean temperature range of (**a**) the Arecaceae and Arecaceae tribes and subfamilies, and (**b**) the most cold-tolerant palm genera. Dashed lines are previously used Arecaceae CMMT thresholds: 2.2 °C from Walther *et al*.^12^, 5 °C from Greenwood and Wing^11^ and 8 °C is the adjustment to Greenwood and Wing^11^ proposed by Royer *et al*.^16^, used in Sluijs *et al*.^14^ and Archibald *et al*.^15^.

The core distribution of the palm family is in subtropical to tropical climates with MAT of 18–28 °C and MART of 0–10 °C (Fig. 2). Importantly, all palm subfamilies and tribes have predominantly tropical climate distributions (Fig. 3a; Table 1). However, a small but significant proportion of the Arecaceae extend into temperate climates, focused on key tribes principally in the subfamily Coryphoideae. The lowest significant MAT when analyzing the Arecaceae as a group is 10.4 °C, but the lowest significant MAT when analyzing individual tribes is 6.9 °C in the subf. Arecoideae, tr. Areceae (Table 1). The lowest coldest quarter mean temperature (CQtrMT) of the Arecaceae is 6.2 °C, 1.9 °C for Areceae and 1.2 °C for subf. Coryphoideae, tr. Trachycarpeae (Table 1). Importantly, in Areceae and Trachycarpeae, the CQtrMT increases to 3.4 °C in both tribes, if the level of significance is raised to 2σ (Table 1). This suggests that CQtrMT of ~3.4 °C is at the extremes of the distribution of these tribes. The maximum MAT at which Arecaceae occur is consistently 30 °C. The lowest maximum MAT is in the subf. Arecoideae tr. Podococceae (MAT ≤ 26.2 °C).

**Table 1:** Results of probability density analysis on the climatic envelope of palms (family, subfamilies and tribes).

	MAT (°C)						MART (°C)						CQtrMT (°C)					
	**0.01**	-**2σ**	**-σ**	**+1σ**	**+2σ**	**0.01**	**0.01**	-**2σ**	-**σ**	**+σ**	**+2σ**	**0.01**	**0.01**	-**2σ**	-**σ**	**+σ**	**+2σ**	**0.01**
Arecaceae	10.4	11.4	14.7	30.0	30.0	30.0	0.2	0.2	0.2	12.8	16.0	17.4	6.2	7.3	10.7	28.4	28.4	28.4
Arecoideae	10.5	11.4	14.5	30.0	30.0	30.0	0.2	0.2	0.3	9.6	11.9	12.8	6.6	8.1	11.7	28.4	28.4	28.4
Calamoideae	15.0	16.3	18.6	28.9	28.9	28.9	0.2	0.2	0.2	10.0	12.5	13.8	10.7	12.1	15.2	27.3	27.3	27.3
Ceroxyloideae	9.1	10.6	13.6	27.4	27.4	27.4	0.4	0.4	0.5	7.2	7.9	9.6	8.6	8.9	12.3	26.8	26.8	26.8
Coryphoideae	8.9	10.3	12.8	30.0	30.0	30.0	0.4	0.4	0.4	17.5	21.1	22.3	3.3	3.9	7.6	28.1	28.3	28.3
Nypoideae	22.3	22.7	25.2	27.6	27.6	27.6	0.2	0.2	0.2	5.0	7.5	8.2	17.6	19.9	22.3	26.9	26.9	26.9
Areceae	6.9	7.9	10.8	28.1	28.1	28.1	0.3	0.3	1.5	11.5	13.8	14.5	1.9	3.4	6.6	25.6	27.0	27.0
Borasseae	19.2	19.7	21.6	29.6	29.7	30.0	0.5	0.5	1.1	8.5	9.7	10.3	15.2	16.1	18.0	28.3	28.3	28.3
Calameae	12.8	14.6	16.8	27.9	27.9	27.9	0.2	0.2	0.2	11.9	15.1	15.7	9.2	9.9	13.1	27.3	27.3	27.3
Caryoteae	15.3	15.3	17.7	27.4	27.4	27.4	0.4	0.4	0.4	14.0	15.0	15.0	9.5	9.5	12.6	26.8	26.8	26.8
Ceroxyleae	9.1	9.9	13.5	26.4	27.4	27.4	0.4	0.4	0.4	7.2	7.6	7.6	8.6	8.9	12.3	24.7	26.6	26.6
Chamaedoreeae	12.3	13.3	15.8	27.5	27.5	27.5	0.2	0.3	0.3	6.5	8.1	8.6	10.1	11.1	13.9	27.0	27.0	27.0
Cocoseae	16.0	16.8	18.9	29.6	30.0	30.0	0.3	0.3	0.3	7.2	8.9	9.6	13.3	14.2	16.6	28.1	28.3	28.3
Corypheae	25.7	25.7	25.7	27.3	27.3	27.3	2.2	3.3	3.3	6.3	7.1	7.1	22.7	22.7	22.7	26.0	26.0	26.0
Cryosophileae	19.9	19.9	20.9	27.4	27.4	27.4	0.6	0.6	0.6	8.2	9.5	10.0	16.1	16.9	18.7	26.6	26.6	26.6
Cyclospatheae	21.3	21.3	24.2	26.6	26.6	26.6	2.8	2.8	3.0	7.9	9.6	9.6	19.7	19.7	20.0	24.9	24.9	24.9
Euterpeae	13.3	14.8	17.4	28.9	28.9	28.9	0.4	0.4	0.5	4.8	5.9	6.4	12.3	14.0	16.1	28.4	28.4	28.4
Geonomeae	11.8	13.2	16.1	28.3	28.3	28.3	0.3	0.3	0.4	6.2	7.8	8.5	11.0	11.7	14.6	27.7	27.7	27.7
Iriarteeae	15.1	15.1	18.1	27.8	28.2	28.2	0.5	0.5	0.5	2.4	2.9	3.1	14.2	14.2	17.1	27.4	27.4	27.4
Leopoldineae	25.4	25.4	25.4	28.4	28.4	28.4	0.8	0.8	0.8	3.1	3.1	3.1	24.4	24.4	24.4	27.0	27.0	27.0
Lepidocaryeae	20.6	21.1	22.4	28.3	28.6	28.9	0.5	0.5	0.5	5.0	5.9	6.5	18.2	18.7	20.5	27.0	27.0	27.0
Manicarieae	25.1	25.1	25.9	27.0	27.0	27.7	0.6	0.6	0.8	2.1	2.1	2.1	24.5	24.5	24.9	26.5	26.5	26.6
Oranieae	20.1	20.1	20.7	26.8	26.8	26.8	0.3	0.3	0.3	6.0	6.2	6.2	17.8	17.8	19.4	26.4	26.4	26.4
Phoeniceae	12.8	12.8	15.0	27.8	29.6	29.6	0.6	0.6	0.6	16.2	20.6	22.0	8.3	8.6	9.5	26.2	28.1	28.1
Phytelephanteae	17.2	19.8	20.2	27.3	27.3	27.3	0.6	0.6	0.6	2.1	2.6	2.8	16.6	19.4	19.5	26.8	26.8	26.8
Podococceae	21.9	21.9	22.3	26.2	26.2	26.2	1.7	1.7	1.7	3.5	4.0	4.0	20.8	20.8	21.0	24.7	24.7	24.7
Reinhardtieae	19.3	20.4	21.1	27.0	27.0	27.0	0.5	0.5	0.8	4.4	5.2	5.5	17.7	19.0	19.7	26.6	26.6	26.6
Roystoneeae	17.7	17.7	21.1	27.5	27.5	27.5	0.7	0.7	0.7	8.4	8.4	11.2	14.8	14.8	19.3	26.8	26.8	26.8
Sabaleae	14.1	15.0	17.0	28.3	28.9	28.9	0.7	0.7	0.7	17.9	20.5	20.5	6.3	6.3	9.8	26.8	28.1	28.1
Sclerospermeae	23.6	23.6	24.1	26.8	26.8	26.8	1.2	1.2	1.2	3.8	3.8	3.8	21.2	21.2	22.3	25.3	25.3	25.3
Trachycarpeae	8.2	10.3	12.2	29.4	29.4	29.4	0.4	0.4	1.1	17.9	20.4	21.9	1.2	3.4	6.3	26.5	27.5	27.5

CMMT calculations show that Trachycarpeae and Areceae have significant occurrences when CMMT ≤ 5 °C (Fig. 3a). Sabaleae (Coryphoideae), Phoeniceae (Coryphoideae) and Ceroxyleae (Ceroxyloideae) have significant occurrences at CMMT ≤ 8 °C (Fig. 3a). As noted before, the level of significance assigned to the outliers of the distribution of Trachycarpeae and Areceae plays an important role, as the 2σ of both tribes is CMMT ≈ 2.2 °C (Fig. 3a). This CMMTmin value is very similar to the threshold of *Trachycarpus fortunei* found by Walther *et al*.^12^.

When considering the 11 genera with occurrences at CMMT ≤ 8 °C separately, the three genera with the coolest range are all in tr. Trachycarpeae (Fig. 3b); *Washingtonia* and *Rhapis* have significant occurrences at CMMT ≤ 5 °C, whereas *Trachycarpus* has a limit of CMMT ≈ 0.2 °C. *Rhopalostylis* and *Linospadix*, the palms belonging to the tr. Areceae with the coldest range, have a limit of CMMT ≈ 4.2 °C and 6.0 °C, respectively. The offset here with the range of the tribe, may be explained by a larger range of MAT and MART in the tribe, providing a more encompassing σ. A small sample size of *Jubaea* included in subf. Arecoideae tr. Cocoseae was too climatically disparate to be representative (Fig. 3a). However, when considering the range of *Jubaea* separately, this genus extends to CMMT ≈ 6.9 °C (Fig. 3b).

Palm climatic envelopes appear highly tribe and subfamily specific (Table 1). Nypoideae is the sole subfamily that is almost exclusively tropical, ranging into subtropical climates at the limit of its distribution (CQtrMT ≥ 17.6 °C). Subfamily Calamoideae is largely tropical to subtropical, but ranges into temperate climates at the limits of its distribution (MAT ≥ 15.0 °C). Subfamilies Arecoideae, Coryphoideae and Ceroxyloideae all extend far into temperate climates (MAT ≥ 10.5, 8.9 and 9.1 °C, respectively). Notably, subfamily Ceroxyloideae appears to occur in areas with a limited seasonal temperature range (MART ≤ 7.9 °C). The tribes Corypheae, Cyclospatheae, Leopoldineae, Manicarieae, Podococceae and Sclerospermeae are strictly tropical. Borassseae, Cocoseae, Cryosophileae, Lepidocaryeae, Oranieae, Phytelephanteae, Reinhardtieae and Roystoneeae also extend into subtropical climates. The tribes Areceae, Calameae, Caryoteae, Ceroxyleae, Chamaedoreeae, Euterpeae, Geonomeae, Iriarteeae, Phoeniceae, Sabaleae and Trachycarpeae extend into temperate climates (Table 1). Notably, many tribes, including Areceae, Ceroxyleae, Chamaedoreeae, Cocoseae, Euterpeae, Geonomeae, Iriarteeae, Phytelephanteae and Reinhardtieae, are more restricted in their MART range than their MAT range.

## Discussion

### The Palm Cold Threshold

The distribution of the Arecaceae is predominantly tropical^22^. The highest diversity and abundance of palms is found in tropical regions where CMMT ≥ 18 °C and MART ≤ 10 °C (Fig. 2). Additionally, all palm tribes have their centres of distribution in the tropics (Supplementary Figures). This is generally well in line with where the main centres of vascular plant diversity in the world are located^26^. Early palm lineage fossils from the Cretaceous were most likely growing in tropical conditions^27^ and palms are interpreted to have diversified and spread out from tropical environments during the late Paleogene and Neogene^20,22^. Despite the preponderance of palms in the tropics, Arecaceae can still occur in fully temperate conditions at MAT ≈ 10 °C, with Areceae and Trachycarpeae even found at MAT = 7–8 °C (Table 1).

Several palm tribes are exclusively tropical, with a CMMT > 18 °C (Fig. 2). Notably, the monotypic *Nypa* (Nypoideae) just exceeds this boundary, occurring at CMMT ≈ 17.0 °C. Nevertheless, Nypoideae has the highest CMMT threshold of all palm subfamilies. A mere six out of 25 tribes (not including Eugeissoneae, Chuniophoeniceae and Pegalodoxeae) are exclusively tropical (Fig. 3a). Our results suggest that palm cold tolerances are highly tribe-specific.

Greenwood and Wing^11^ suggested that CMMT ≈ 5 °C is the lower threshold of palm distribution. This agrees well with our statistically derived threshold of the Arecaceae of CMMT ≈ 5.2 °C, when excluding the outliers of the whole family distribution. However, when considering the range of the palm tribes separately, it is evident that some of these outliers of the Arecaceae distribution are within the significant range of the Areceae and the Trachycarpeae. Whereas our data includes records of Areceae down to CMMT ≈ 0.8 °C and Trachycarpeae down to CMMT ≈ 0.1 °C, these occurrences are at the extreme end of the probability distribution (<0.01; ≫-2σ) and we would argue not a true reflection of the limit of viable reproducing palm populations (Fig. 3a). The genus with the coolest range in the Areceae tribe, *Rhopalostylis*, has a significant range down to CMMT ≈ 4.2 °C, which is above the 2σ of the tribe Areceae range (CMMT = 2.3 °C). It is likely that the large variation within the Areceae distribution, in both MAT and MART, causes σ to be large and inclusive of occurrences that are statistical outliers with the more conservative σ of *Rhopalostylis*. We should therefore assume that the CMMT threshold of Areceae is at 2.3–4.2 °C.

The lower threshold of *Trachycarpus* (CMMT ≈ 0.2 °C) agrees with that of the tribe Trachycarpeae (CMMT ≈ 0.1 °C). This is well below the CMMT = 2.2 °C threshold for *Trachycarpus* found by Walther *et al*.^12^. Problematic in this case is the coordinate precision of the extreme range of *Trachycarpus*, which at its extreme northern limits occurs in incised valleys with a strong microclimate. Even small coordinate imprecisions could place *Trachycarpus* in a highland, rather than a valley. Moreover, it is questionable if the global climate models we used here to simulate climatic range are precise enough to capture microclimates. The CMMT = 0.1 °C threshold of Trachycarpeae is therefore questionable and we recommend the 2σ value of CMMT ≈ 2.2 °C, in agreement with Walther *et al*.^12^. The -3.2 °C CMMT threshold for *Trachycarpus fortunei* found by Fang *et al*.^23^ in China indicates that this species can be successful in cultivation at CMMT < 2.2 °C, but available data shows that the species does not naturalize at these temperatures^6,7,12^. *Trachycarpus* vegetative tissue freezes at relatively low temperatures (>-14.0 °C) and can recover even after frost damage, but ground frost of <-8 °C destroys the roots of *Trachycarpus fortunei*, with seeds killed at -2.5 °C^7^. We can therefore assert that sustained frost is fatal to Trachycarpeae and that the diurnal temperature range should be large enough to go above ground frost temperatures. Finally, *Rhapis* and *Washingtonia* (Trachycarpeae) both have a lower CMMT threshold of 2.5 °C, suggesting that cold-tolerance in Trachycarpeae is not confined to *Trachycarpus* (Fig. 3b) and supporting the lower CMMT threshold in Trachycarpeae of ~2.2 °C.

Cocoseae is shown to be exclusively subtropical to tropical, with a lower CMMT threshold of 12.5 °C and lower MAT threshold of 16.0 °C (Fig. 3b, Table 1). However, this eliminates the range of *Jubaea*, which can occur down to CMMT ≈ 6.9 °C (Fig. 3b). Though the monospecific genus *Jubaea* is considered threatened within its native range^28^ and its climatic range is strongly anomalous within the Cocoseae (Supplementary Figure 1m), it should not be excluded from the lower CMMT threshold estimate of the Cocoseae. The lower CMMT threshold of Ceroxyleae (7.7 °C) is offset somewhat from *Ceroxylon*, which still occurs at CMMT < 5 °C (Fig. 3a,b). This is probably due to preponderance of Ceroxyleae in the tropics, especially *Ravenea*, which is confined to Madagascar and the Comoros islands. *Ceroxylon* is present at high altitudes (>3000 m) in the tropical Andes, explaining its low MAT and CMMT, as well as the relatively restricted MART range of Ceroxyleae (≤7.9 °C).

Sabaleae, comprised only of *Sabal*, extends into temperate latitudes (principally *S. minor*) and accordingly has a relatively low CMMT threshold (5.3 °C). Despite a relatively high CMMT threshold in comparison to *Trachycarpus*, *Sabal minor* is one of the most frost-hardy palms, only suffering widespread frost damage at <-13.5 °C^7^. The range of *Phoenix* (Phoeniceae) is somewhat problematic, predominantly because of the widespread cultivation of *Phoenix dactylifera*, but also *P. carariensis*, *P. reclinata*, *P. roebelenii*, *P. rupicola* and *P. sylvestris* (Supplementary Table 1). When considering the native range of *Phoenix*, the lower CMMT threshold is at 7.4 °C. However, geospatial data in the native range of the most transplanted species, *P. dactylifera* (date palm), is poor and the second most transplanted species, *P. canariensis* (Canary Island date palm) is endemic to the Canary Islands, but widely cultivated around the Mediterranean. It is therefore unclear if our lower threshold may be an underestimate. *Phoenix* has similar frost tolerance to *Washingtonia*^7^.

### The influence of seasonality

**Figure 4:**
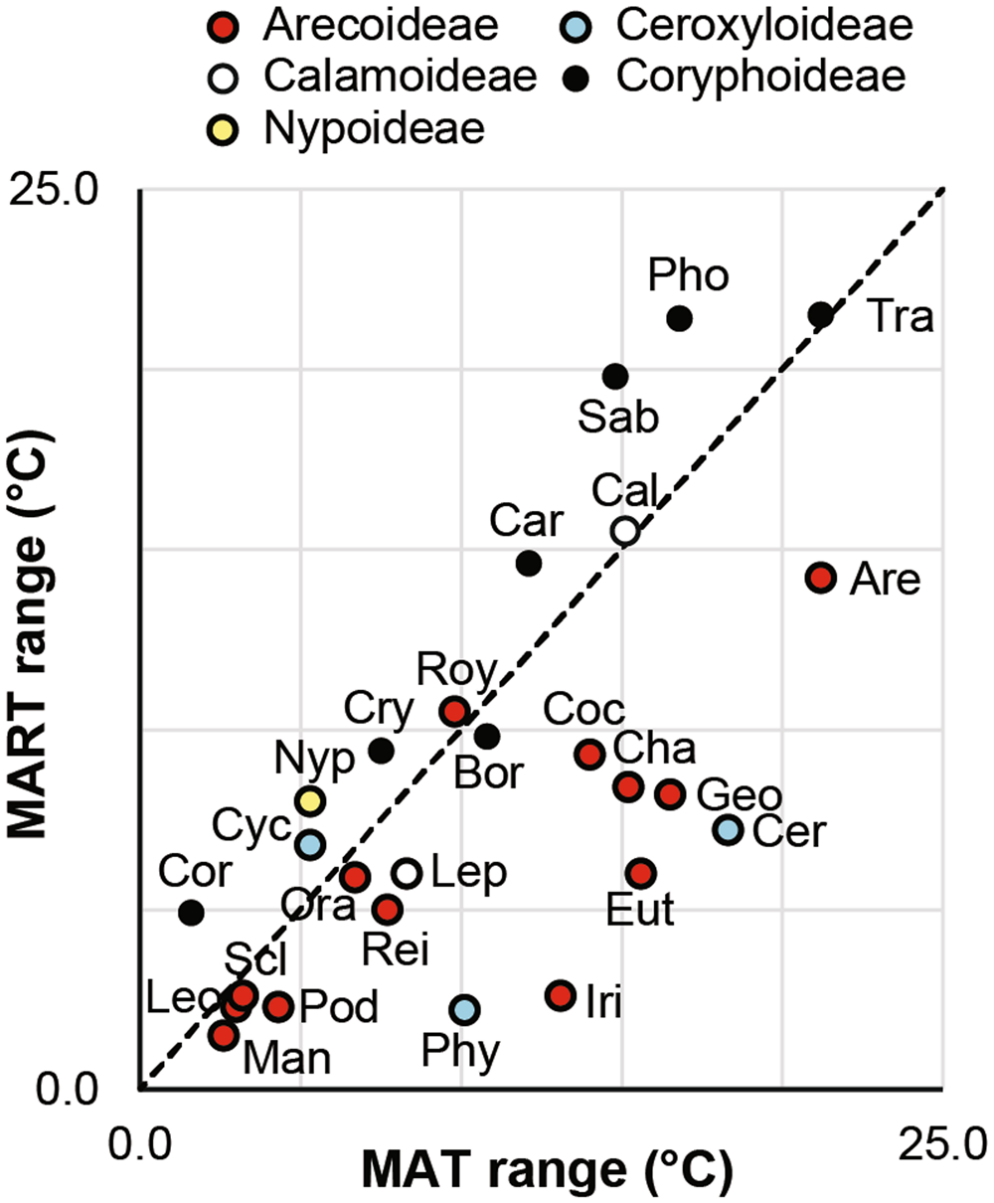
MATmax – MATmin vs MARTmax – MARTmin of palm tribes. The dashed line indicates where MATmax – MATmin = MARTmax – MARTmin.

In general, our data show that the MAT range of a palm tribe increases with the MART range (Fig. 4). This is indicative of the latitudinal range of particular tribes, as the MAT decreases and MART increases with increasing latitude. A cluster of palm tribes in Fig. 4 have a relatively high MAT and MART range: Trachycarpeae, Phoeniceae, Sabaleae, Areceae, Calameae and Caryoteae. Notably, Arecoideae appear to tend towards a MAT range that exceeds the MART range, whereas Coryphoideae are generally the converse (Fig. 4). Furthermore, the results presented here suggest that in some palm tribes MART may play a more important role in determining distribution than MAT. In 8 tribes, the MAT range strongly exceeds the MART range (Fig. 4). This is notably the case for Areceae, Ceroxyleae, Euterpeae, Geonomeae, Chamaedoreeae and Cocoseae. In the case of Iriarteeae and Phytelephanteae the MART range is close to 0 °C, with a MAT range of ~13 and ~10 °C, respectively. This suggests that these tribes are restricted to ecosystems in the tropics with very little intra-annual variation. Limited seasonality may be more important in determining the distribution of these tribes than the temperature around which this seasonality occurs^29^. Climates with limited MART are usually at tropical latitudes and/or in strongly ocean-moderated climates. These palm tribes are distributed along an altitudinal gradient, probably occurring in lowland and montane tropical rainforest.

There are less extreme cases of palm tribes with a large MART range, such as Phoeniceae, Sabaleae and Caryoteae, without a correspondingly large MAT range (Fig. 4). This is probably due to a combination of a lack of freezing tolerance in palms^7^ and that the highest MART ranges occur at temperate latitudes. Phoeniceae and Sabaleae appear to deviate most toward a higher MART range than MAT range. The native range of Phoeniceae is the Mediterranean semi-arid climates of the Middle East, Persia and North Africa, where the air holds little moisture that could moderate seasonal temperature swings. The native range of Sabaleae extends into the southeast United States, where climates are not strictly continental, but experience cold winters, as cold air moves southward across the North American continent.

### Palaeoclimatic implications

Palms are common in the fossil record, first appearing around the mid-Cretaceous^30^. They are recovered commonly as pollen^31^, foliage^32,33^, wood^34^, phytoliths^35^ and fruits^27,36–38^. Flowers are relatively rare due to their delicate structure^30,39^. During the early Eocene, palms enjoyed a near-cosmopolitan range, including in the Arctic and the Antarctic^14,18,40^. The presence of palms at polar latitudes is puzzling as the physiology of modern palms suggests that winter dormancy is unlikely^8^. Latent heat loss in an absence of insolation during winter would drive temperatures at the poles down, causing sub-freezing temperatures that palms could not withstand. Increased equator to pole heat transfer^41^ as well as increased cloud cover^5,42^ in combination with high global CO_2_^43,44^ probably kept polar regions relatively warm in Eocene winters. The Eocene Arctic at 72–85°N had an inferred CMMT ≥ 8 °C^14,40^ based on the presence of palms adjusted for high *p*CO_2_, whereas Antarctica at ~65°S had a CQtrMT ≥ 10 °C^18^ based on the presence of a multitude of cold-intolerant species, including palms.

**Figure 5:**
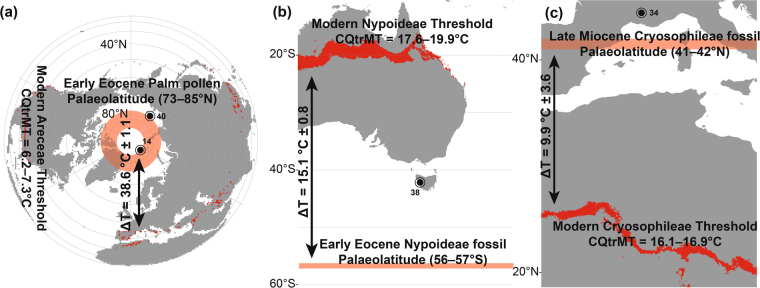
Examples of using fossil palm occurrences as palaeoclimatic indicators. All figures generated using ArcGIS^58^. The red area indicates where the specific CQtrMT tolerance of this (sub-) family or tribe can be found today and the pink line indicates the latitude at which the fossils were deposited. The ΔT indicates the difference in CQtrMT at that latitude during the time of deposition and today, (**a**) palm pollen from the early Eocene high Arctic^14,40^, (**b**) Nypoideae fruit from the early Eocene of Tasmania^38^ and (**c**) Cryosophileae wood from the late Miocene of southern France^34^.

Royer *et al*.^16^ found using chamber experiments, that the freezing sensitivity of palms increases by 1.5–3 °C in high *p*CO_2_ (~800 ppm). Early Eocene hyperthermals, such as the PETM or EECO, have *p*CO_2_ estimates of >800 ppm^43,44^. The CMMT threshold obtained here for Trachycarpeae would put the early Eocene CMMT at 3.7–5.2 °C. However, since diversification and radiation of Trachycarpeae did not occur until the Miocene^45^, the more conservative CMMT of the entire palm family is more appropriate here, putting early Eocene CMMT at 6.7–8.2 °C. This is >30 °C warmer than the Arctic CMMT is today and therefore in support of the enhanced Eocene poleward heat flux and increased cloud cover necessary to keep the poles warm during winter^5,41,42^. However, Royer *et al*.^16^ asserted a 1.5–3 °C increase in freezing sensitivity of plants based on chamber experiments. Interpreting chamber effects is problematic^46^, and raised CO_2_ levels in growth chambers are known to cause high levels of aborted, or malformed, stomata^47^. Aborted stomata are thought to be indicative of a failure of leaf expansion or cell division to adjust the distance of stomatal initials^48^, suggesting that the leaf is non-competitive, or ill adjusted, to the circumstances^49^. It is important to note that natural selection drives adaptation, including to differing levels of atmospheric CO_2_, and that a leaf generation, or a single plant generation, does not represent a natural experiment^50^. Increased freezing sensitivity in high CO_2_ chamber experiments may therefore be indicative of a plant poorly adapted to the high levels of CO_2_. Further experiments would be needed to show if plants grown in a high-CO_2_ chamber are experiencing the deleterious effects of frost and not high-*p*CO_2_. For now, we assume a minimum CMMT of 5.2 °C, derived from the modern distribution of the Arecaceae family, corresponding to within 1σ of terrestrial climate from the early Eocene based on leaf physiognomy^51^. This would imply that early Eocene winter temperatures in the Arctic were 38.6 ± 1.1 °C warmer than today (Fig. 5a).

Nearest living relative analyses in the fossil record rely on the assumptions that the modern-day distribution of a taxon is representative of its full climatic potential and that this range is dependent on evolutionary conservative traits^52^. The confidence of this assumption becomes less robust with increasing fossil age, as phylogenetic relationships become more disparate from modern. However, the Arecaceae are highly speciose with some palm tribes diverse in genera and geographically widespread^25^ and the phylogeny and origination/diversification ages well-studied^20,22,25,45^, thus fulfilling multiple criterion for strong nearest living relative climate proxies^53^. Caution is required in using individual palm genera as palaeoclimatic indicators; e.g., the monospecific genus *Jubaea* – if *Jubaea* were extinct, we may assume that Cocoseae did not occur at CMMT < 12.6 °C (Fig. 3a). However, *Jubaea* CMMTmin = 6.9 °C (Fig. 3b). In this study, we attempt to compensate for these problems by including a large modern representative dataset and assuming an inclusive minimum threshold range for minimum estimates. Using palm tribe-specific bioclimatic threshold values we can estimate temperature minimums for the palm fossils that have been successfully assigned to a tribe. For example, Carpenter *et al*.^38^ identified *Nypa* from early Eocene western Tasmania (palaeolatitude: 56–57°S) and suggested that this was indicative of a tropical mangrove environment, which corresponds to the Nypoideae threshold at CMMT = 17.0–19.4 °C or CQtrMT = 17.6–19.9 °C (Table 1, Fig. 3a). Compared to the modern-day climate at 56–57°S, this would mean that CQtrMT during the early Eocene at this latitude was 15.1 ± 0.8 °C warmer (Fig. 5b). Modern day regions with the same CQtrMT are found predominantly at 20°S, or at 25°S in coastal situations. This would put early Eocene Tasmania just within the range of a subtropical climate (CMMT ≤ 18.0 °C), but very likely tropical (CMMT ≥ 18.0 °C). Thomas and De Fransceschi^34^ identified Cryosophileae wood remains in the late Miocene of southern France (palaeolatitude: 41–42°N). The Cryosophileae CQtrMT threshold would suggest that late Miocene CQtrMT = 16.1–16.9 °C, 9.9 ± 3.6 °C warmer than CQtrMT of that region at the same latitude in modern times (Fig. 5c).

Palm fossils are relatively abundant but assigning them to a tribe or genus can be problematic^30,32,33^. Further, the identification of palms from fossil pollen is potentially questionable, with some fossil pollen unambiguously identified as ‘palm’, with other palm-like pollen variously assigned to Areaceae or other unrelated plants^14,15,18,30,31,40^. However, the three examples above show the potential of using the presence of palm fossils as threshold indicators. In some cases, where adequate taxonomic information is available, a tribe- or subfamily-specific threshold value can be used. However, in assemblages with non-tribe or –subfamily specific taxonomic assignments a threshold value for Arecaceae can still be used. There is potential with several palm fossil organs for tribe-specific taxonomic assignments. Palm leaf fossils should be identified using cuticle if present, as the epidermis often provides genus-diagnostic characters^54–56^; however, great strides have been made in the taxonomic classification of palm wood^34^ and palm pollen^31^.

## Methods

To obtain a comprehensive dataset of palms worldwide, we extracted a databank of 729,696 georeferenced Arecaceae records from gbif.org on February 27, 2017, using the most current palm classification^25^. This dataset represents an amalgamation of records from several publishing authorities (Supplementary Table 2) and is unbiased in its recordkeeping. However, we manually filtered the dataset according to several criteria:

- Entries with no species data were removed.
- Entries with poor or non-specific geospatial data were removed. Poor geospatial data can be identified if the geodetic coordinates are strongly rounded, assumed, or depict a location that is not on dry land.
- Redundant entries were removed. Particularly when geodetic data is back-entered, a single location may be chosen to represent a sampled population of multiple individuals. This can lead to several hundreds of individual records with identical geodetic coordinates. The rationale for only having a single record representing a location is that when an already small dataset is composed mostly of multiple records from a single location, the climatic probability function becomes highly slanted towards the climate of that location.

The dataset was then manually filtered for cultivated individuals. This was done by:

- Identifying palm species that are (1) popular as ornamentals, (2) cultivated crops and/or (3) an invasive species. Within the geodetic data, distinguishing ornamentals, cultivars and invasive species from each other is often problematic. Therefore, no distinction was made between them, despite the possibility of an invasive or naturalized species occurring within its preferred environment.
- Identifying the countries that are included in the natural range of the species. The output file of gbif.org includes information of the country in which the plant was recorded. This allows us to exclude occurrences in countries that are not included in the natural range of the species.

We identified 56 palm species as having a problematic range (Supplementary Table 1). Of the original dataset of 729,696 georeferenced Arecaceae records, this rigorous filtering process resulted in 21,323 occurrences that were used (Fig. 1). For the Chilean wine palm (*Jubaea chilensis*) there were only two recorded occurrences in its native range. However, because of its importance for climatic range estimates as the most temperate species of the tribe Cocoseae, we supplemented the database with 11 recorded populations from Gonzalez *et al*.^28^. All geodetic coordinates of these occurrences were queried for MAT, summer and winter mean temperature (WQtrMT and CQtrMT) using the gridded climate model of Hijmans *et al*.^57^ in ArcGIS^58^. Mean annual range of temperature (MART) was obtained by calculating the mean difference between WQtrMT and CQtrMT. Finally, we obtained CMMT by establishing a linear transform function between CQtrMT and CMMT based on climate data of 410 stations in North America and Oceania (Supplementary Table 34), where:

1 $$CMMT=1.03\times CQtrMT-1.17$$

This method of calculating introduces errors of up to 2.5 °C for very cold sites where CQtrMT <-5 °C, as in some climates the CMMT can be much cooler than the CQtrMT. However, error quantification suggests that this higher error only occurs in highly continental sites, with long subfreezing winters; not places where palms occur. To accommodate the error in other areas we calculated a 95% confidence interval of 0.66 °C (Supplementary Table 34).

Manual filtering eliminates a part of the potential for climatic outliers (Fig. 1). However, the method that we use here still allows for bias. Primarily because the dataset is strongly dependent on organizations, such as government agencies (herbaria, universities, conservation bodies), that make their records available and the degree of data availability can differ strongly between countries. As an example, New Zealand has two native palm species that we consider here, with 2713 records, whereas Papua New Guinea has 129 species of palm that we consider here, with 556 records. This may result in a bias towards the temperate New Zealand species, even though these are much less diverse and not necessarily more abundant. Secondly, we eliminated outliers manually based on the occurrence latitudinal range of a palm species and which countries the species is native in. Therefore, the possibility exists that occurrences are included that are outside the native range of the plant, within a country that it is native in. For example, the palm could be planted on a mountain, whereas its native range is at sea level in the same country. Finally, manual filtering cannot account for the reliability of the geodetic information; data can be incorrectly entered into the database or not recorded accurately at the time of collection (e.g., for older records made prior to the widespread use of handheld GPS systems). We therefore applied a probability density analysis to calculate the likelihood of each individual record being representative. Probability density analysis assumes a normal distribution in the climatic range of each group considered. Assuming a normal distribution is advantageous in this type of analysis because it reduces the risk of bias by poor geospatial coverage. It also allows for querying individual occurrences for their significance in determining the climatic range of a group.

We performed probability density analysis on three taxonomic ranks: family level, subfamily level and tribe level. This provided a significant sample size in most cases. The Eugeissoneae, Chuniophoeniceae and Pegalodoxeae tribes had three, eight and ten recorded occurrences, respectively, that were left after manual filtering. Therefore, these tribes were not further considered. To determine the probability of a data point (x) being representative of the range we combined probability density of both MAT and MART.

2 $${f}_{x}=(\frac{1}{\sqrt{2{{\sigma }_{MAT}}^{2}\pi }}{e}^{\frac{{({X}_{MAT}-{\mu }_{MAT})}^{2}}{2{{\sigma }_{MAT}}^{2}}})\times (\frac{1}{\sqrt{2{{\sigma }_{MART}}^{2}\pi }}{e}^{\frac{{({X}_{MART}-{\mu }_{MART})}^{2}}{2{{\sigma }_{MART}}^{2}}})$$

Because this calculation would yield different probability densities within each group, the probability for each data point was compared to the maximum probability likelihood within a group to determine the relative probability of this data point in constituting the climatic range of the group.

3 $${f}_{x(relative)}=\frac{{f}_{x}}{{f}_{max}}$$

We postulate that a data point with a relative probability of f_x(relative)_ < 0.01 is insignificant in the climate range of the group. We determined the range of one (f_x(relative)_ = 0.157) and two standard deviations (f_x(relative)_ = 0.023) from the occurrence within a group with f_max_ to constrain the core distribution of a group. This method is preferable over assigning percentiles as cutoffs for significant climatic range, as we determine outliers based on the relative deviation from the mean climatic range of the group. This also allows us to determine if a group of data does not have any outliers, or may be prone to being influenced by outliers. In addition, this method can consider the significance of a datapoint along multiple environmental gradients.

When referring in the results and discussion to tropical, subtropical or temperate climates, we follow the climatic classification of Belda *et al*.^59^. Climates are considered tropical if CMMT > 18 °C. Climates are subtropical if ≥ 8 months have average temperatures of >10 °C and a CMMT of <18 °C. Since we do not consider monthly temperatures, we here refer to a climate as subtropical if MAT is >16 °C and CMMT is <18 °C. In Belda *et al*.^59^, climates with 4–7 months of temperatures >10 °C are considered temperate. Here, we refer to temperate climates as MAT < 16 °C.

### Data availability

All data used in this study is made available in the Supplementary Information.

## Electronic supplementary material

Supplementary figures

Dataset 1
