# Supplementary material for: The relation between global palm distribution and climate
Source: Sci Rep. 2018 Mar 16;8:4721. doi: 10.1038/s41598-018-23147-2 (PMC5856843; doi:10.1038/s41598-018-23147-2)
Supplement: Supplementary file 1 — Supplementary figures [file 41598_2018_23147_MOESM1_ESM.pdf]

Supplementary figures

The relation between global palm distribution and climate

Tammo Reichgelt, Christopher K. West, David R. Greenwood

Supplementary figure 1: Mean annual temperature (MAT) vs mean annual range of temperature (MART) for all occurrences used in the analyses for this manuscript of the palm family, subfamilies and tribes. (a) Arecaceae, (b) Arecoideae, (c) Calamoideae, (d) Ceroxyloideae, (e) Coryphoideae, (f) Nypoideae, (g) Areceae, (h) Borasseae, (i) Calameae, (j) Caryoteae, (k) Ceroxyleae, (l) Chamaedoreeae, (m) Cocoseae, (n) Corypheae, (o) Cryosophileae, (p) Cyclospatheae, (q) Euterpeae, (r) Geonomeae, (s) Iriarteeae, (t) Leopoldinideae, (u) Lepidocaryeae, (v) Manicarieae, (w) Oranieae, (x) Phoeniceae, (y) Phytelephanteae, (z) Podococceae, (aa) Reinhardtieae, (ab) Roystoneeae, (ac) Sabaleae, (ad) Sclerospermeae, (ae) Trachycarpeae.

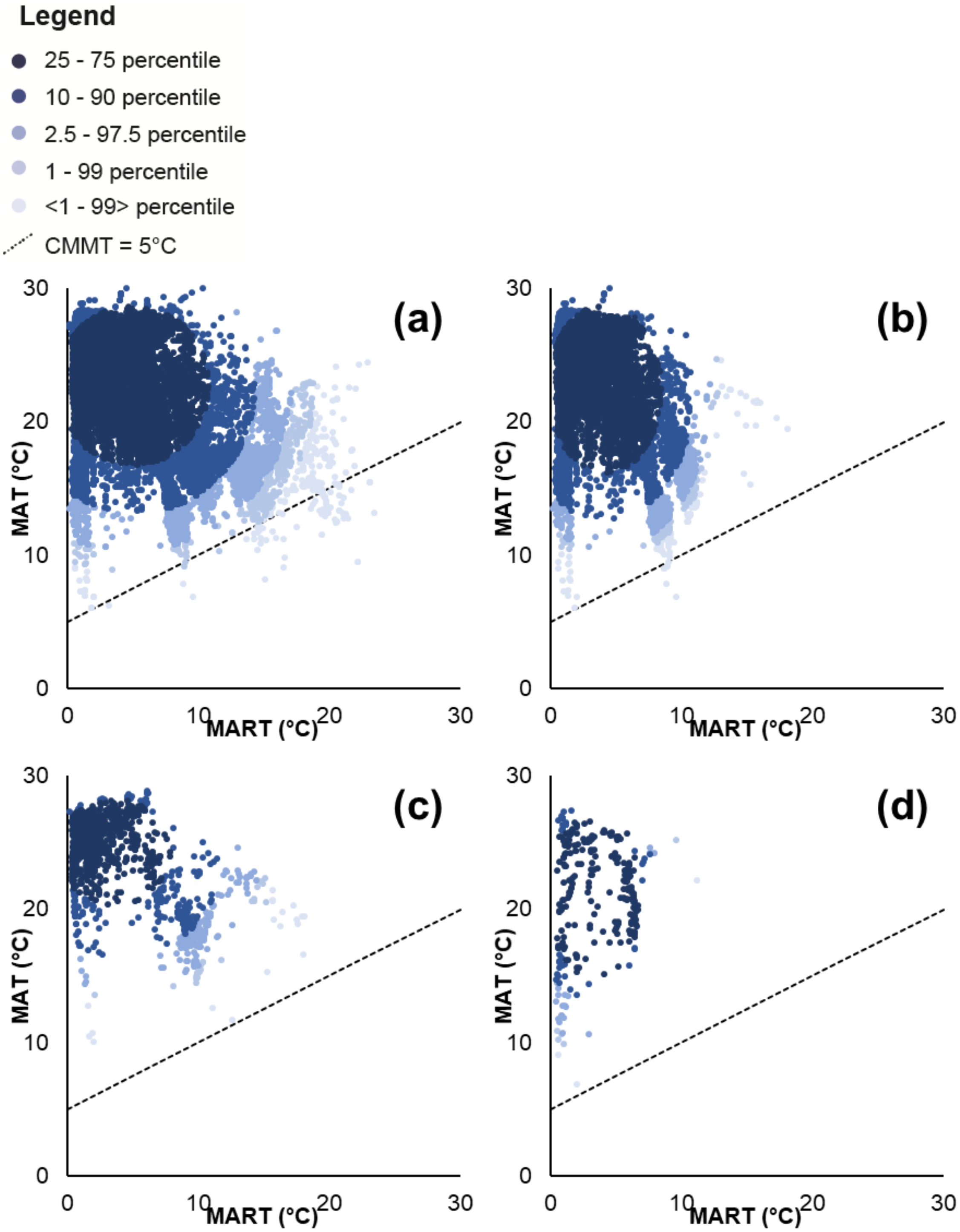

Legend
25 - 75 percentile
10 - 90 percentile
2.5 - 97.5 percentile
1 - 99 percentile
<1 - 99> percentile
CMMT = 5°C
(a)
(b)
(c)
(d)
MAT (°C)
MART (°C)
0
10
20
30

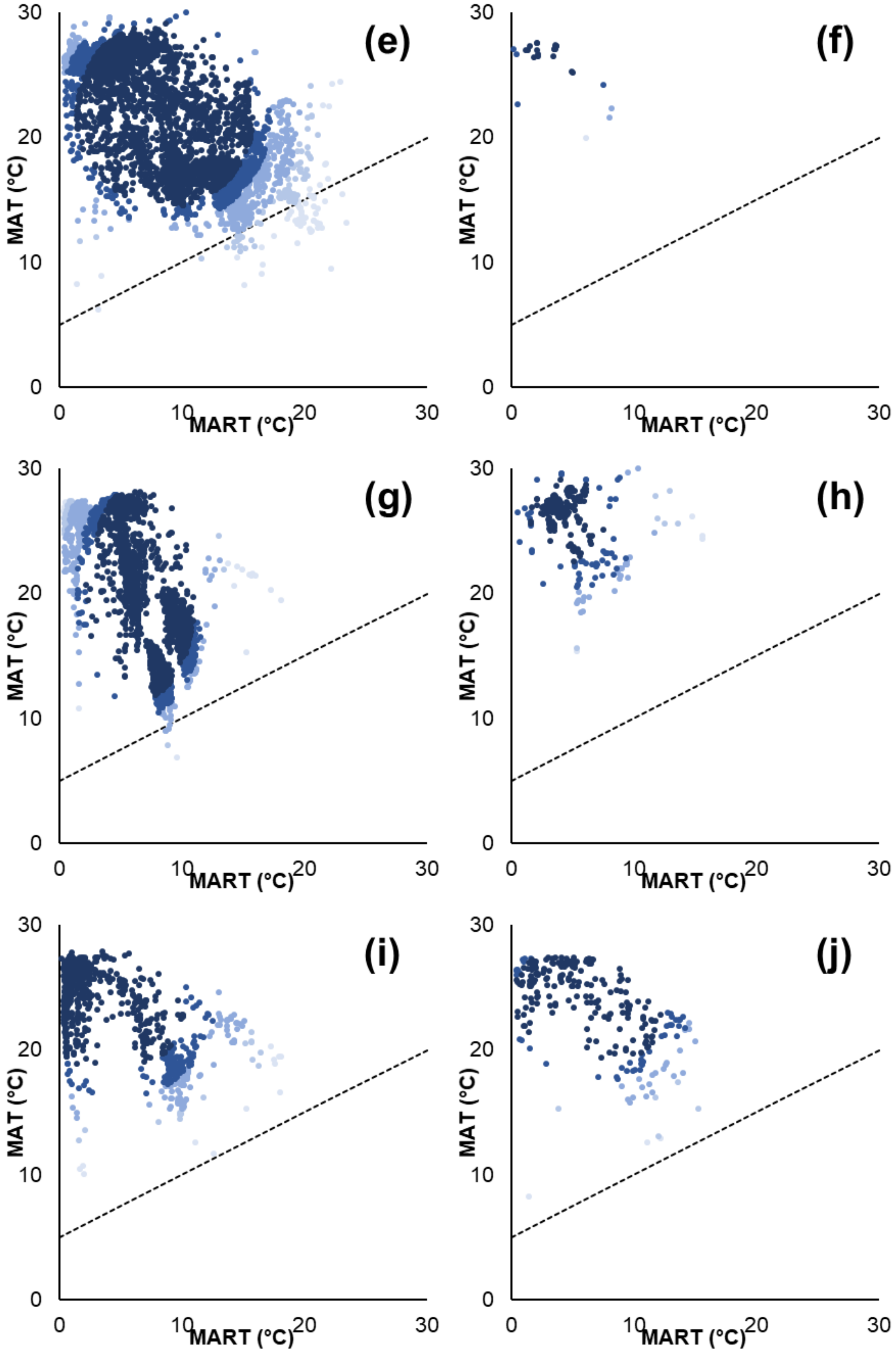
(e)
MAT (°C)
MART (°C)
(f)
MAT (°C)
MART (°C)
(g)
MAT (°C)
MART (°C)
(h)
MAT (°C)
MART (°C)
(i)
MAT (°C)
MART (°C)
(j)
MAT (°C)
MART (°C)
0
10
20
30

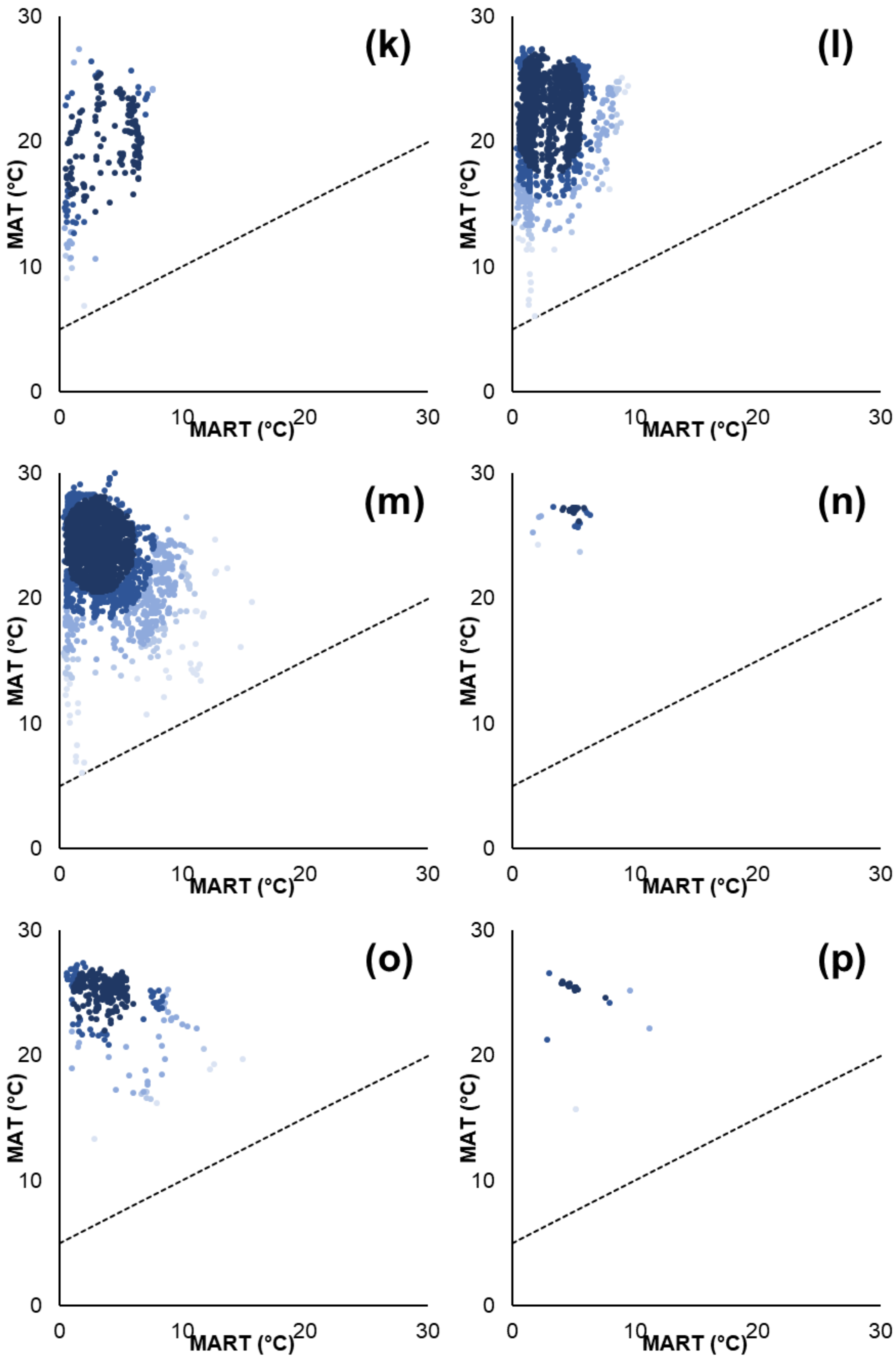
(k)
(l)
(m)
(n)
(o)
(p)
MAT (°C)
MART (°C)
0
10
20
30

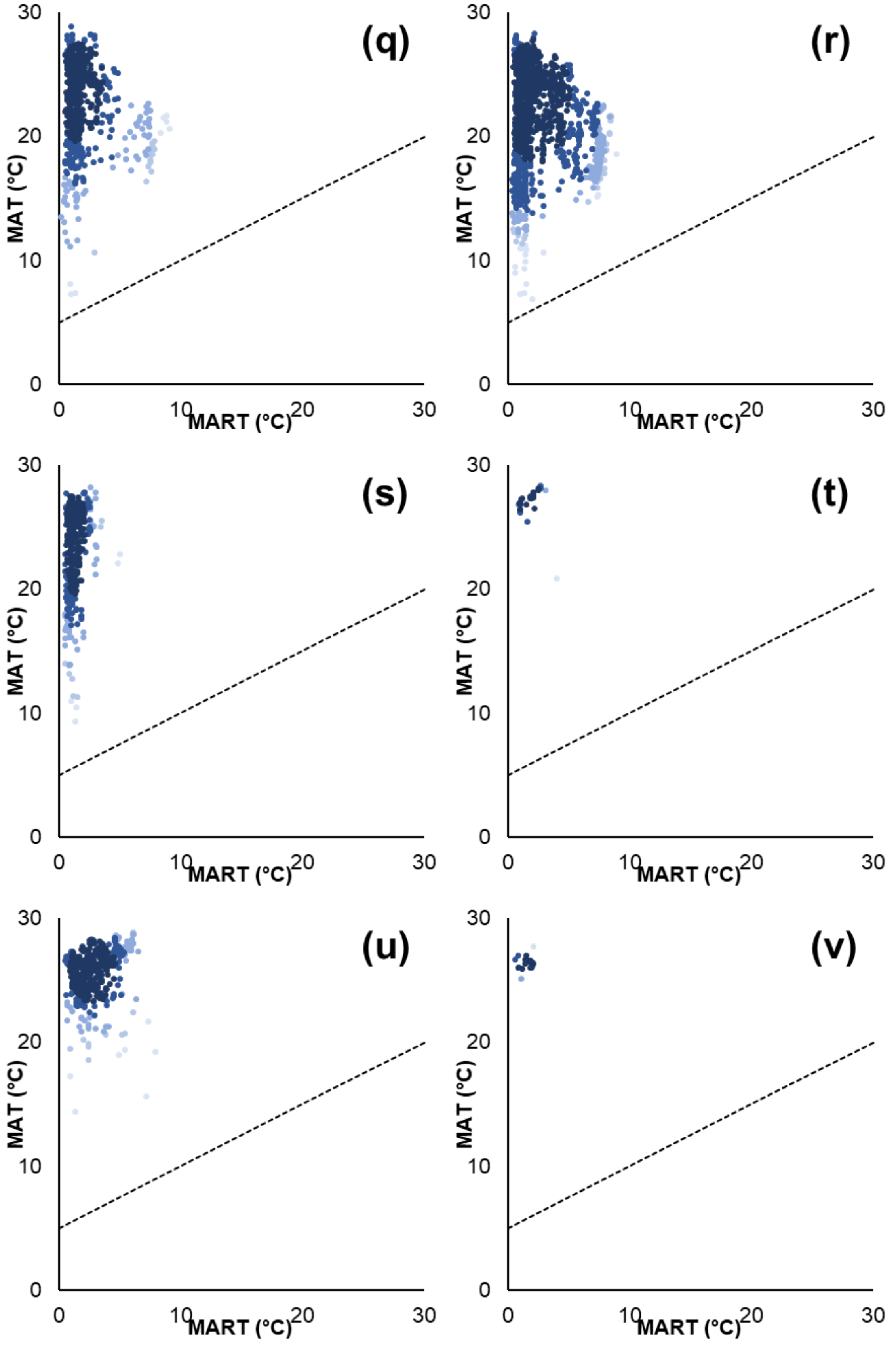
(q)
(r)
(s)
(t)
(u)
(v)
MAT (°C)
MART (°C)
0
10
20
30

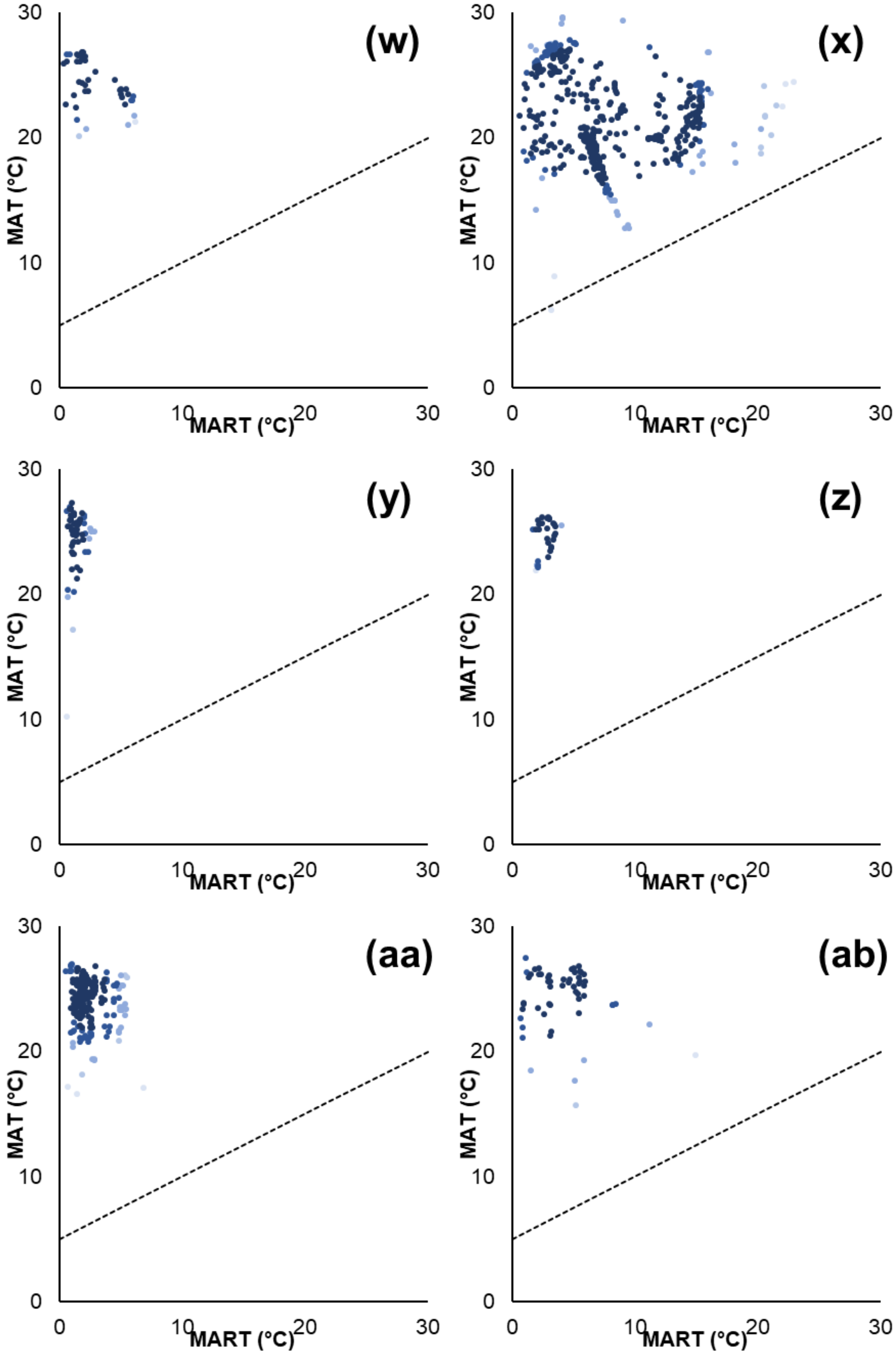
(w)
(x)
(y)
(z)
(aa)
(ab)
MAT (°C)
MART (°C)
0
10
20
30

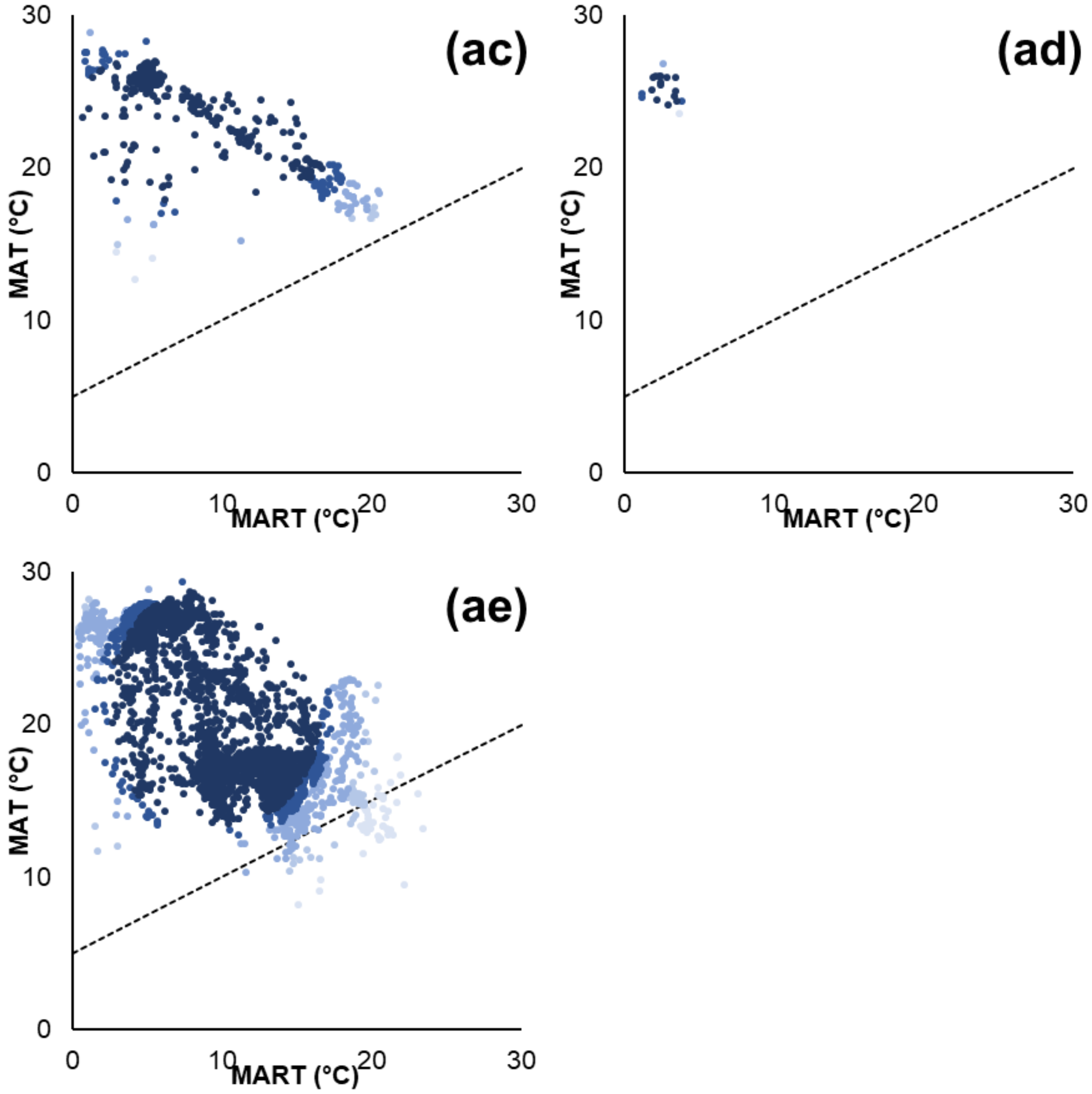
(ac)
(ad)
(ae)
MAT (°C)
MART (°C)
0
10
20
30
